# Black Rice (*Oryza sativa* L.) Fermented with* Lactobacillus casei* Attenuates Osteoclastogenesis and Ovariectomy-Induced Osteoporosis

**DOI:** 10.1155/2019/5073085

**Published:** 2019-02-19

**Authors:** Young Min Lee, In Sook Kim, Beong Ou Lim

**Affiliations:** ^1^Department of Applied Life Science, Graduate School of Konkuk University, Chungju 27478, Republic of Korea; ^2^Department of Life Science, College of Biomedical and Health Science, Konkuk University, 268 Chungwondaero, Chungju-si, Chungcheongbuk-do 27478, Republic of Korea; ^3^Research Institute of Inflammatory Diseases, Konkuk University, Chungju 27478, Republic of Korea

## Abstract

The aim of the present study was to investigate the antiosteoclastogenic effects of black rice (*Oryza sativa L.*) fermented with* Lactobacillus casei* (LAB) in RANKL-induced RAW macrophage cells and its antiosteoporosis activity against ovariectomy-induced osteoporosis in rats. LAB extract (LABE) treatment attenuated receptor activator of nuclear factor-kappa B (NF-*κ*B) ligand-induced osteoclastic differentiation in RAW cells by inhibiting intercellular reactive oxygen species generation and downregulating the activation of mitogen-activated protein kinases and NF-*κ*B, leading to the downregulation of c-Fos and expression of nuclear factor of activated T cells c1. This consequently suppressed the expression of osteoclast-specific genes including those for cathepsin K, tartrate-resistant acid phosphatase, calcitonin receptor, and integrin *β*3. Oral administration of LABE protected against ovariectomy-induced bone loss by significantly inhibiting bone architecture alterations and improving serum bone turnover markers in ovariectomized rats. The findings suggest that the antiosteoporotic activity of LABE may be derived from its antiosteoclastic and anti-bone-resorptive activities. LABE has potential as a promising functional material or substrate to prepare protective agents for osteoporosis and osteoclast-mediated bone diseases.

## 1. Introduction

Osteoporosis is a chronic skeletal disorder characterized by the weakening of bones, atrophy, and degradation of the bone microarchitecture, resulting in increased bone fragility and susceptibility to fracture [1]. Living bone is composed of rigid dynamic tissues that undergo constant remodeling to maintain homeostasis [2], which is mediated by a delicate balance between bone resorption by osteoclasts and bone formation by osteoblasts [3]. Enhanced osteoclast function and formation result in excessive bone resorption that causes osteoclastogenesis, ultimately leading to bone diseases including osteoporosis [4–7]. Therefore, osteoclasts are important targets for the treatment of pathological bone loss disorders.

Osteoclasts are multinucleated giant cells formed from monocytes/macrophages. They are derived from hematopoietic progenitors through the stimulation of macrophage colony-stimulating factor (M-CSF) and receptor activator of nuclear factor-kappa B (NF-*κ*B) ligand (RANKL). The M-CSF and RANKL cytokines are essential for osteoclastogenesis. M-CSF is essentially expressed by osteoblasts, whereas RANKL expression by osteoblasts is stimulated by several osteotropic factors [8]. Binding of RANKL to the receptor activator of NF-*κ*B (RANK) modulates the interaction of tumor necrosis factor receptor-associated factor 6 (TRAF6) with RANK. The RANKL/RANK signal is then transmitted to downstream signaling cascades, including NF-*κ*B and mitogen-activated protein kinase (MAPK) pathways, such as c-Jun N-terminal kinase (JNK), extracellular signal-regulated kinase (ERK), and p38 pathways in osteoclast precursor cells [9]. Subsequently, TRAF-mediated signaling pathways activate NF-*κ*B, c-Fos, and nuclear factor of activated T cells c1 (NFATc1), which are the transcription factors principally responsible for osteoclast differentiation [9]. In particular, NFATc1, a principal regulator of osteoclast differentiation, induces the expression of a number of osteoclast-specific genes including tartrate-resistant acid phosphate (TRAP), calcitonin receptor, cathepsin K, and integrin *β*3 [10], leading to the development of mature osteoclasts.

The bran fraction of black rice (*Oryza sativa* L.) is a rich source of antioxidant compounds, including anthocyanins and tocopherol [11]. Anthocyanins, such as cyanidin-3-O-glucoside and peonidin-3-O-glucoside, are the main effective constituents of black rice and they help protect arteries, prevent oxidative DNA damage, and inhibit the formation of cell damaging free radicals [12–14]. According to a recent report, black rice extract exerts osteogenic activities by stimulating osteoblast differentiation [14]. It has also been shown that daily oral administration of black rice extract at 200 mg/kg for 8 weeks prevents decreases in bone density and strength in ovariectomized rats [15].

Numerous health benefits are claimed to be derived from fermented products, and these products have consequently garnered significant interest [16]. Microbial fermentation of food products facilitates the production of biologically active metabolites and enhances the bioavailability of food-derived phytochemicals and nutrients [17]. Previously, it was reported that black rice bran fermented with fungi and lactic acid bacteria could be utilized as a health-functional food and pharmaceutical agent [18]. Additionally, several prebiotics cause changes in intestinal microorganisms, and these changes are closely related to increased bone mineral density and strength, as well as increased calcium absorption in animal models of inflammation [19–21].

In this study, we evaluated the inhibitory effect of the 70% ethanol extract of black rice fermented with* Lactobacillus casei* (LAB) on osteoporosis in the presence of reactive oxygen species (ROS) and when osteoclast differentiation was inhibited. We determined the anthocyanin compounds in LAB extract (LABE) and investigated their effects on RANKL-induced osteoclast differentiation in RAW 264.7 macrophage cells and their preventive effects against ovariectomy-induced osteoporosis in rats.

## 2. Materials and Methods

### 2.1. Preparation of the Fermented Black Rice Extract

*Lactobacillus casei* (*L. casei*) cultures were grown and maintained on Lactobacilli MRS agar medium (Difco, Sparks, MD, USA). Lactobacilli MRS broth medium (Difco) was used to propagate the organisms prior to their use in fermenting the black rice. Black rice was autoclaved at 125°C for 15 min after soaking overnight in 5% (w/v) aqueous aronia (*Aronia melanocarpa*) solution.* L. casei* were then inoculated into the black rice and cultured at 37°C for 3 days. The fermented black rice was dried in hot air at 40°C and then ground. Extraction was performed using 70% ethanol at room temperature for 24 h. This extraction process was repeated three times. Subsequently, the extracts were pooled and filtered through Whatman no. 2 filter paper and concentrated using a rotary vacuum evaporator (EYELA, Tokyo, Japan) at 40°C. The concentrated LABE was lyophilized and stored at -20°C until use. The yield (Y) was calculated using the following equation: Y (%) = (total extracted sample weight/total dry sample weight) × 100.

### 2.2. High-Performance Liquid Chromatography (HPLC) Analysis

Identification of the anthocyanin compounds in LABE was performed using HPLC (Dionex UltiMate 3000 Series, Thermo Fisher Scientific, Waltham, MA, USA) equipped with a Syncronis C18 analytical column (5 *μ*m, 250 mm × 4.6 mm) (Thermo Fisher Scientific). The analytical conditions for HPLC included a column temperature of 40°C, a flow rate of 1 mL/min, an injection volume of 10 *μ*L, and a wavelength of 520 nm. Mobile phases were composed of 0.1% trifluoroacetic acid (A) and acetonitrile (B), where the gradients were 10%–15% B at 0–5 min, 18% B at 5–10 min, 20% B at 10–15 min, 25% B at 15–20 min, 30% B at 20–25 min, and 10% B at 25–35 min. Peak identification of anthocyanins was confirmed by the retention time and the spectrum of the standards. Anthocyanin standards, including cyanidin-3,5-diglucoside, cyanidin-3-glucoside, cyanidin-3-rutinoside, and peonidin-3-glucoside, were purchased from Sigma-Aldrich (St. Louis, MO, USA). The data were collected using the Chromeleon™ Chromatography Management System (Version 6.80; Thermo Fisher Scientific).

### 2.3. Determination of Total Phenolic and Flavonoid Content

The total phenolic content was determined using the Folin-Ciocalteu method [22]. Gallic acid was used to generate the standard curve. The total phenolic content was expressed as gallic acid equivalents (GAE) in mg per 100 g dry mass. The total flavonoid content was measured by the aluminum colorimetric method described previously [23]. The standard curve was made using catechin. The total flavonoid contents were expressed as catechin equivalents in mg per 100 g dry mass. All samples were analyzed in triplicate.

### 2.4. DPPH Radical Scavenging Activity

The DPPH (2,2-diphenyl-1-picrylhydrazyl) radical scavenging activities of LABE were analyzed as described previously [24]. Sample and standard solutions of 80 *μ*L were mixed with 80 *μ*L of 0.2 mM DPPH solution. The reaction mixture was kept in the dark with continuous shaking for 30 min at room temperature. Finally, the absorbance of the mixture was measured at 517 nm.

### 2.5. ABTS Radical Scavenging Activity

ABTS [2,2′-azinobis-(3-ethylbenzothiazoline-6-sulfonic acid)] radical scavenging activity of LABE was determined using a previously described method [25] with slight modifications. ABTS cations were produced by reacting 7 mM ABTS in water with 2.45 mM potassium persulfate (1:1), and these were stored in the dark at room temperature for 12-16 h before use. The ABTS cation solution was then adjusted with 0.1 M phosphate-buffered saline (PBS, pH 7.4) to an absorbance of 0.70 ± 0.02 at 734 nm. After the addition of 200 *μ*L of LABE and ascorbic acid to 800 *μ*L of diluted ABTS cation radical solution, the absorbance was measured 5 min after the initial mixing at 734 nm.

### 2.6. Determination of Reducing Power

The ferric reducing power of the sample extract was estimated by a previously reported method [26] with slight modification. First, 0.5 mL of 0.2 M sodium phosphate buffer (pH 6.6) and 0.5 mL of 1% (w/v) potassium ferricyanide (Fe^3+^) were mixed with 0.5 mL of each sample and standard (0.25–4.0 mg/ml) to form potassium ferrocyanide (Fe^2+^). The mixture was incubated in a water bath for 20 min at 40°C. After incubation, 1 mL of each mixture was added to 0.5 mL of 10% trichloroacetic acid and centrifuged for 10 min at 4°C. Following this, 0.5 mL of the supernatant was mixed with 0.5 mL distilled water and 0.1 mL of 0.1% w/v FeCl_3_. The absorbance was measured at 700 nm.

### 2.7. Cell Culture

RAW 264.7 murine macrophage cells (Korean Cell Line Bank, Seoul, Korea) were cultured in Dulbecco's modified Eagle's medium (DMEM) containing 10% heat-inactivated fetal bovine serum, penicillin (100 units/mL), and streptomycin (100 *μ*g/mL) in a 37°C incubator in an atmosphere of 5% CO_2_ in air.

### 2.8. Determination of Intracellular ROS

The levels of intracellular ROS were determined through the oxidation of 2′7′-dichlorodihydrofluorescein diacetate (H_2_DCF-DA; Sigma-Aldrich, St. Louis, MO, USA) to fluorescent 2′,7′-dichlorofluorescein (DCF; Sigma-Aldrich) by hydroperoxides. RAW 264.7 cells (5 × 10^4^ cells/well) were seeded into the wells of a 96-well plate and incubated for 24 h at 37°C. The cells were treated with the extract at various concentrations (10–1000 *μ*g/mL) and incubated at 37°C for a further 24 h. After incubation, 10 *μ*M H_2_DCF-DA was added to the cells and the cells were incubated for 40 min. Then, 50 *μ*M hydrogen peroxide (H_2_O_2_) or 50 ng/mL RANKL was added and reacted at 37°C for 1 h. The fluorescence was detected by excitation at 485 nm and emission at 530 nm.

### 2.9. Tartrate-Resistant Acid Phosphatase (TRAP) Activity Assay

To measure TRAP activity, RAW 264.7 cells were seeded in 96-well plates (5 × 10^3^ cells/well). After 24 h of incubation, the medium was replaced with differentiation medium (*α*-MEM) containing 50 ng/mL recombinant mouse RANKL (PeproTech, Rocky Hill, NJ, USA). After cell differentiation for 3 days, the cells were lysed by adding lysis buffer (0.1% Triton X-100 containing 80 mM sodium tartrate and 90 mM citrate, pH 4.8) and the cell lysates were incubated with 80 *μ*L of substrate solution (20 mM p-nitrophenyl phosphate) for 20 min at 37°C. Finally, 0.5 N NaOH (40 *μ*L) was added to stop the enzymatic reaction. The absorbance was measured at 405 nm using a UV spectrophotometer (Tecan Austria GmbH, Salzburg, Austria).

### 2.10. TRAP Staining

TRAP staining was performed using a TRAP staining kit (no. 387A; Sigma-Aldrich) according to the manufacturer's instructions. Images of TRAP-positive multinucleated cells were captured using a microscope (CKX41; Olympus, Tokyo, Japan) equipped with a digital video camera (eXcope T500, Olympus, Tokyo, Japan).

### 2.11. Western Blot Analysis

RAW 264.7 cells seeded at a density of 8 × 10^3^ cells/well in 6-well plates were cultured with DMEM for 24 h at 37°C in a 5% CO_2_ incubator. After 24 h, the medium was replaced with *α*-MEM containing 50 ng/mL RANKL and the sample extract. After 3 days, the cells were lysed with PRO-PREP buffer (iNtRON Biotechnology, Seongnam, Korea) to extract the expressed protein. Nuclear protein was extracted with NE-PER nuclear and cytoplasmic extraction reagents (Thermo Fisher Scientific). The concentration of the protein extract was determined using a protein assay with bovine serum albumin (BSA) as the standard. Proteins were resolved via SDS-PAGE and transferred to a nitrocellulose membrane. The membranes were blocked in 5% BSA and incubated overnight at 4°C with the primary antibodies specific for the targeted molecules. The membranes were then incubated with the corresponding secondary antibodies, and the targeted proteins in the membrane were visualized by enhanced chemiluminescence (ECL) reagents. The relative intensities of the bands were visualized and analyzed using the ChemiDoc XRS+ imaging system (BIO-RAD Laboratories, Hercules, CA, USA) equipped with Image Lab software.

### 2.12. Experimental Animals and Treatments

Eight-week-old female Sprague-Dawley (SD) rats (270–290 g) were purchased from Oriental Bio (Seongnam, Korea). The rats were acclimated for one week prior to experiments. All animals were housed in a specific pathogen-free animal room at Konkuk University in accordance with the guidelines of the Principles for the Care and Use of Animals in the Field of Physiological Sciences, published by the Physiological Society of Korea, and maintained at constant temperature (25 ± 2°C) and humidity (55%  ± 5%) on a 12-h light/dark cycle. Female rats were ovariectomized (OVX) and allowed a recovery period of 2 weeks. The experimental animals were allocated to four groups (N = 10 per group): sham-operated (sham), OVX-control (OVX), OVX 17-*β*-estradiol-treated (E2), and 500 mg/kg LABE-treated OVX (LAB). After confirming the occurrence of osteoporosis at 8 weeks, LABE and 17-*β*-estradiol diets were provided from 9 to 16 weeks. LABE treatments were orally administered daily, and estrogen was subcutaneously injected once a week with depot preparations of estradiol valerate (200 *μ*g/kg, Estradiol Depot 10 mg, Jenapharm, Jena, Germany).

### 2.13. Bone Micro-Computed Tomography (Micro-CT) Scanning

Bone micro-CT analyses of the right proximal femur were conducted using a model 1078* in vivoμ*-CT scanner (SkyScan, Kontich, Belgium) with an isotropic resolution of 18 *μ*m. The scanning system was calibrated at 70 kV, 85 lA, and 1000 projections per 180 degrees with a 350 ms integration time. From the stack of cross-section images, a volume of interest (VOI) containing only cancellous bone was extracted for morphometric analysis using CT Analyzer V 1.11.0.0 software (SkyScan). The VOI began 1 mm from the lower end of the growth plate and extended distally for 110 cross sections (2 mm high). For morphometric analysis, the structural parameters calculated over each VOI of cancellous bone by three-dimensional (3D) analysis (using the aforementioned CT Analyzer software) included bone volume fraction (BV/TV), trabecular thickness (Tb.Th), trabecular number (Tb.N), direct trabecular separation (Tb.Sp), and bone mineral density (BMD) [27]. The average attenuation coefficient of the trabecular bone tissue was determined for all measurements by a protocol provided by SkyScan.

### 2.14. Bone Histomorphometric Analysis

Right femurs were decalcified in 5% formic acid in distilled water for 1 week, dehydrated with water, and embedded in paraffin. The paraffin sections were deparaffinized and stained (hematoxylin & eosin (H&E) and TRAP stains). Sections exhibiting the widest marrow cavity near the growth plate of the metaphysis of the femur were selected for further histological processing and histomorphometric measurements. Histomorphometric measurements were conducted using an Optiphot-2 microscope (Nikon, Tokyo, Japan) connected to an RGB camera and a personal computer housing Lucia G 4.51 software (Laboratory Imaging, Prague, Czech Republic) at 100× magnification.

### 2.15. Biochemical Analysis of Serum Chemistry

The serum levels of calcium (ab102505 calcium detection assay kit; Abcam, Cambridge, UK) were measured using assay kits according to manufacturer's instructions. Briefly, 10 *μ*L samples of diluted serum adjusted to 50 *μ*L with distilled H_2_O were added to each reaction well. Chromogenic reagent (90 *μ*L) was added to each well containing the samples. Sixty microliters of Calcium assay buffer was added to each well and the plate was incubated at room temperature for 5-10 min protected from light. The absorbance was measured at 575 nm. The concentrations of serum calcium were calculated using a standard curve.

The serum levels of alkaline phosphatase (ALP) were measured using assay kits (ab83369, Alkaline Phosphatase Assay Kit; Abcam) according to the manufacturer's instructions. Briefly, 80 *μ*L of sample dilutions, 50 *μ*L of 5 mM p-nitrophenyl phosphate (pNPP) solution, and 10 *μ*L of ALP enzyme solution were added sequentially to the reaction wells and the plate was incubated at 25°C for 60 min protected from light. The reaction was halted by adding 20 *μ*L stop solution, and then the absorbance was measured at 405 nm. ALP activity was calculated using a standard curve.

### 2.16. Statistical Analyses

All data were analyzed using Prism version 6.05 (GraphPad Software, La Jolla, CA, USA). Data for animal experiments are expressed as means ± standard error of difference (SED).* In vitro* data are presented as the mean ± standard deviation (SD) from three independent experiments. All statistical analyses were performed using ANOVA followed by Dunnett's multiple-comparison test. Differences between groups were considered statistically significant at* P* < 0.05.

## 3. Results

### 3.1. HPLC Analysis

**Figure 1:**
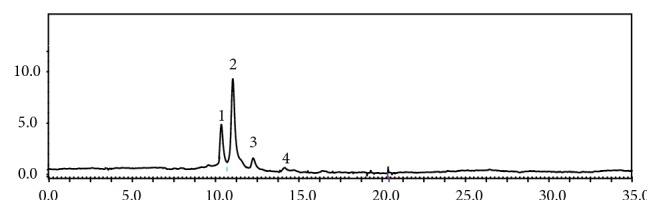
HPLC chromatogram of anthocyanin compounds present in LABE. The analytical conditions of HPLC were as follows: column temperature, 40°C; flow rate, 1 mL/min; injection volume, 10 *μ*L; and wavelength, 520 nm. Mobile phases were composed of 0.1% trifluoroacetic acid (A) and acetonitrile (B) with the following gradients: 10%–15% B in 0–5 min, 18% B in 5–10 min, 20% B in 10–15 min, 25% B in 15–20 min, 30% B in 20–25 min, and 10% B in 25–35 min.

In this study, the chemical composition of LABE was analyzed using an HPLC system with detection at 520 nm (Table 1 and Figure 1). The four anthocyanin compounds were identified as cyanidin-3,5-diglucoside (0.0858 mg/g), cyanidin-3-glucoside (1.0592 mg/g), cyanidin-3-rutinoside (0.0123 mg/g), and peonidin-3-glucoside (0.0421 mg/g). As shown in Table 1, of the four anthocyanins, cyanidin-3-glucoside (peak 2) was the most abundant in the extract. Black rice is remarkably high in anthocyanin pigments, which account for its violet color [28]. Anthocyanins are also found in high concentrations in dark fruits, such as blueberries, blackberries, dark grapes, and dark cherries. With more efficient and stronger antioxidant activity, black rice may be a better source of anthocyanins than blueberries [29]. Previous findings identified different anthocyanins in different black rice cultivars, which possessed different antioxidant capacities. In each case, antioxidant activity was positively correlated with total anthocyanin content [28, 30, 31].

### 3.2. Total Phenolic and Flavonoid Contents and Extraction Yield

Total phenolic and flavonoid compound levels correlate well with antioxidant activity [30]. In our study, total phenolic and total flavonoid contents were assessed in LABE (Table 2) and were found to be 168.7 ± 2.70 mg GAE per 100 g dry mass and 214.38 ± 16.77 mg catechin equivalent per 100 g of dry mass, respectively. The percentage yield (w/w) of 70% ethanol LABE was 7.44%.

### 3.3. Free Radical Scavenging Activity

**Figure 2:**
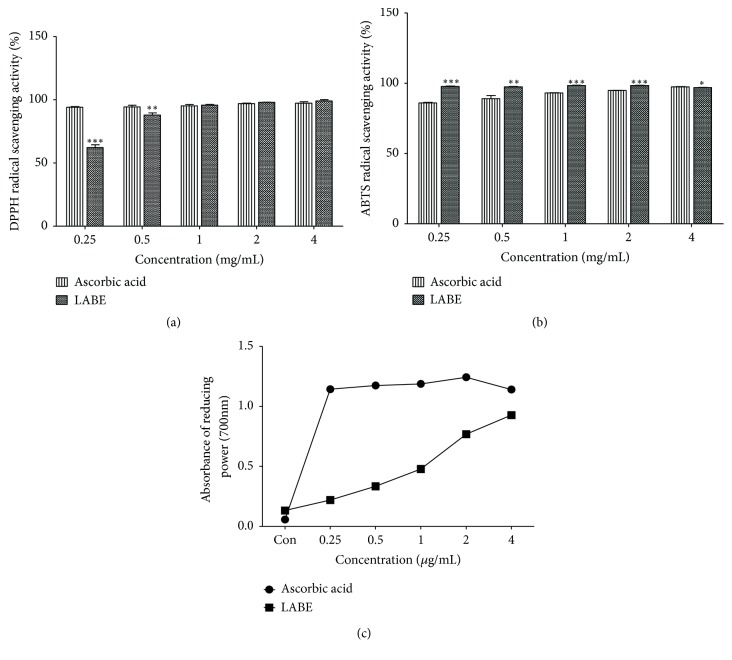
Antioxidant activity of LABE. (a) DPPH radical scavenging activity of LABE. (b) ABTS radical scavenging activity of LABE. The values are the mean ± SD from three independent experiments *∗ P *< 0.05, *∗∗ P* < 0.01, and *∗∗∗ P *< 0.001 versus control group. Ascorbic acid is used as a control. (c) Reducing power activity of LABE. The data are expressed as means (n = 3).

Antioxidant capacities of LABE were measured using DPPH and ABTS radical scavenging activities, as well as the reducing power assay. Ascorbic acid was chosen as a positive control. As shown in Figure 2(a), LABE showed strong antioxidant activities in the three antioxidant assays. LABE exhibited a high, dose-dependent DPPH free radical scavenging activity. Notably, LABE possessed the same DPPH radical scavenging activity as that of ascorbic acid at 1.0 mg/mL. ABTS radical scavenging activity of LABE was significantly higher than that of ascorbic acid, which was used as a reference substance at the same concentration.

### 3.4. Reducing Power Activity

Reducing power is generally associated with the presence of reductones (antioxidants), which exert antioxidant activity by disrupting the free radical chain reaction by donating a hydrogen atom [32]. Reducing power was measured using the FRAP assay, which determines the antioxidant activity of a sample by measuring color changes associated with the transformation of Fe^3+^ to Fe^2+^. The reducing power of LABE was notable and increased with increasing concentrations of LABE (0.25–4.0 mg/mL), suggesting that LABE possessed high antioxidant capacity. For example, at a concentration of 4 mg/mL, the reducing power of LABE was similar to that of ascorbic acid.

### 3.5. Inhibition of Intracellular ROS

ROS are generated as a normal cellular metabolic byproduct in living cells and are considered hazardous at high concentrations [33, 34]. Oxidative damage can suppress osteogenesis [35], and excessive ROS levels induce osteoblast/osteocyte apoptosis [34]. Osteoclasts are particularly sensitive to oxidative stress [36]. Therefore, low levels of ROS can promote osteoclast differentiation and bone resorption [36–38].

**Figure 3:**
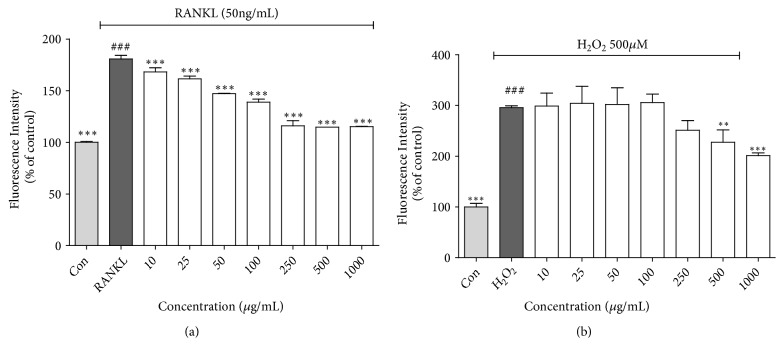
Effects of LABE on ROS inhibition. (a) Inhibition of H_2_O_2_-induced ROS generation in RAW 264.7 cells by LABE. (b) Inhibition of RANKL-induced ROS generation in RAW 264.7 cells by LABE. After 24 h of sample treatment, 10 *μ*M of H_2_DCF-DA was added to the cells and incubated for 40 min, followed by addition of 50 *μ*M H_2_O_2_ or 50 ng/mL RANKL for 1 h. All data are presented as the mean ± SD of three independent experiments performed with n = 3. Compared are positive control (not treated group) versus negative control (only H_2_O_2_ or RANKL-treated group, ###* P *< 0.001) and negative control versus sample treated group (*∗P* < 0.05, *∗∗ P* <0.01, and *∗∗∗ P <*0.001).

Recently, it was reported that ROS act as intracellular signal mediators in upstream signaling pathways associated with osteoclast activation and osteoclast differentiation. In these pathways, RANKL induces NADPH oxidase-derived ROS, a process that is essential for osteoclast differentiation [33, 39, 40]. Given this, the sustained generation of ROS is associated with induction of osteoclast differentiation and with osteoporosis. Therefore, we evaluated the effect of LABE on the production of hydrogen peroxide- (H_2_O_2_-) induced and RANKL-induced ROS. H_2_O_2_ has been used as an oxidative stress inducer for the evaluation of antioxidant ability in many studies. Here, we investigated the effect of LABE on the production of ROS induced by H_2_O_2_. ROS production in RAW 264.7 cells was higher following treatment with 50 *μ*M H_2_O_2_ than it was in the untreated control group. Treatment with LABE at concentrations of 250 to 1000 *μ*g/mL significantly decreased ROS generation in a dose-dependent manner (Figure 3(a)). RANKL-mediated signaling in osteoclast precursor bone marrow macrophages can be intensified by the intracellular ROS generated by RANKL [41]. Additionally, RANKL-induced ROS act as intracellular mediators, activating the ERK signaling pathway and subsequently causing osteoclast differentiation and activation [33]. We evaluated the inhibitory effect of LABE on RANKL-induced ROS generation. Intracellular ROS generation increased by up to 180.7% in RAW 264.7 cells following RANKL treatment compared to levels observed in the control group (Figure 3(b)). The production of intracellular ROS was significantly decreased in a dose-dependent manner by treatment with LABE at a concentration range of 10 to 1000 *μ*g/mL. These results suggest that LABE can effectively reduce intracellular ROS production.

### 3.6. Effect of LABE on Osteoclast Differentiation

**Figure 4:**
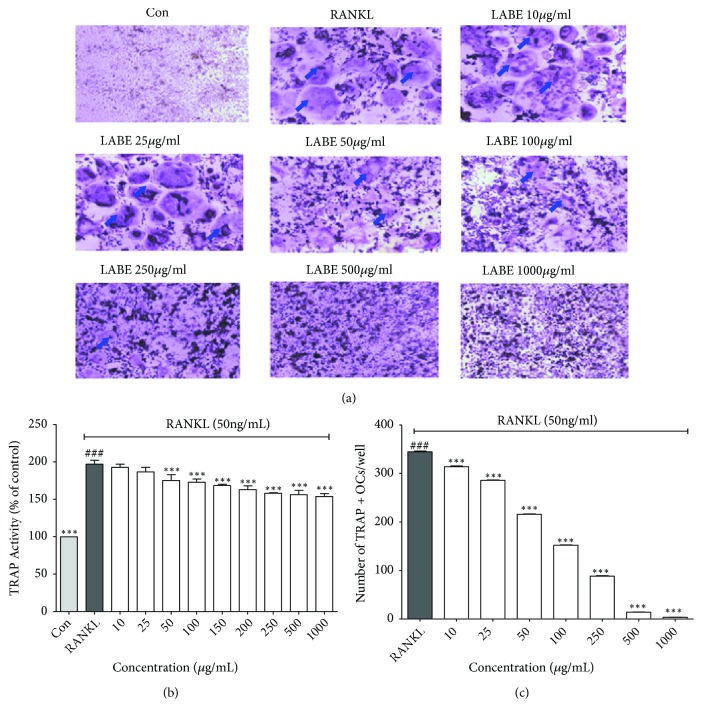
Inhibitory properties of LABE on differentiation and TRAP activity. (a) Inhibitory effects of LABE on RANKL-induced round-shaped osteoclast formation (blue arrow) in RAW 264.7 cells. Cells were exposed to RANKL (50 ng/mL) in the presence or absence of LABE for 5 days. The cells were fixed and stained using a leukocyte acid phosphatase (TRAP) kit. (b) Inhibitory effects on TRAP activity of RANKL-induced osteoclasts in RAW 264.7 cells by LABE. Cells were exposed to RANKL (50 ng/mL) in the presence and absence of LABE for 3 days. TRAP activity was measured by TRAP solution assay. (c) Number of TRAP-positive multinucleate osteoclasts. Con: positive control (which was not treated), RANKL: negative control (which was treated with only RANKL), sample treated group: RANKL+ sample. TRAP-positive multinucleated osteoclasts were visualized at 40× magnification using light microphotography. All data are presented as the mean ± SD of three independent experiments performed with n = 3. Statistical analyses were performed by comparing Con (positive control, not treated) versus RANKL (negative control, treated with only RANKL, ###* P *< 0.001) and RANKL versus sample treated group (*∗∗∗P < *0.001).

RAW cells can differentiate into macrophages or osteoclasts in the presence of stimulators, such as RANKL [42]. RANKL is a member of the tumor necrosis factor (TNF) family and is essential for the differentiation and activation of osteoclasts [43]. In RANKL-treated RAW cells, RANKL binds to RANK to activate the intracellular pathway, thereby promoting the differentiation of osteoclast precursor cells into TRAP-positive multinuclear osteoclasts [33]. TRAP is highly expressed by osteoclasts and activated macrophages. We therefore evaluated the effect of LABE on RANKL-induced osteoclast differentiation and TRAP activity by means of a TRAP activity and staining assay. The RAW cells were cultured for 3 days in the presence of RANKL (50 ng/mL) with or without 10 to 1000 ppm LABE, and the differentiated cells were evaluated. The results of the TRAP activity and staining assay showed that LABE significantly suppressed the differentiation of osteoclast precursor cells in TRAP-positive multinuclear osteoclasts and TRAP activity in a dose-dependent manner (10-1000 ppm; Figures 4(a), 4(b), and 4(c)). TRAP activity increased by up to 196.98% compared to activity observed in the control following RANKL treatment (Figure 4(b)) and the number of TRAP-positive multinucleate osteoclasts also increased (Figures 4(a) and 4(c)). Both TRAP activity and the number of TRAP-positive multinucleate osteoclasts decreased following addition of LABE.

### 3.7. Effect of LABE on MAPK Activation

**Figure 5:**
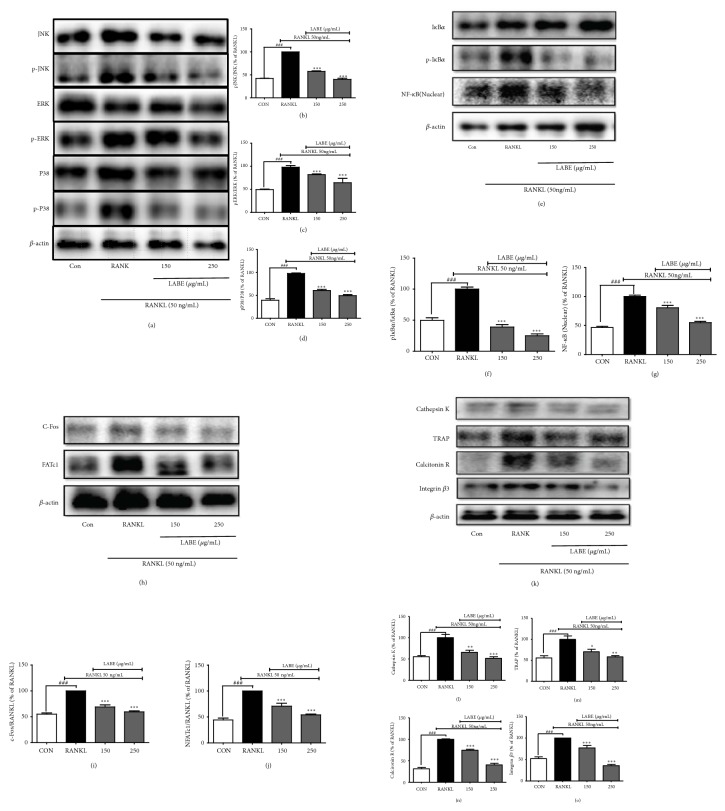
Inhibition of MAP kinase, NF-*κ*B activation, and gene expression of c-Fos, NFATc1, cathepsin K, TRAP, calcitonin receptor, and integrin *β*3 during RANKL-induced osteoclast formation in RAW 264.7 cells by LABE. (a–d) LABE altered the phosphorylation of the MAPKs (JNK, ERK, and p38). (e–g) LABE altered the phosphorylation of the I*κ*B*α* and inhibited nuclear translocation of I*κ*B*α* and NF-*κ*B. (h–j) LABE inhibited the expression of c-Fos and NFATc1. (k–o) Inhibitory effects on gene expression of cathepsin K, TRAP, calcitonin receptor, and integrin *β*3 during RANKL-induced osteoclast in RAW 264.7 cells by LABE. Cells were exposed to RANKL (50 ng/mL) in the presence and absence of LABE for 3 days. The protein expression levels were determined by western blot analysis, and *β*-actin was used as loading control. RANKL-treated group was considered as 100% for densitometric analysis. All data are presented as the mean ± SD of three independent experiments performed with n = 3. *∗ P *< 0.05, *∗∗ P* < 0.01, and *∗∗∗ P < *0.001 versus RANKL-treated group; and ###* P *< 0.001 versus Con group. Con: positive control (not treated); RANKL: negative control (treated with only RANKL).

MAPK family members, which include ERK, JNK, and p38 MAPK, are essential for osteoclast differentiation [44, 45] and are activated by RANKL through the MAPK signaling pathway in osteoclast precursor cells [5, 32]. The activated MAPK signaling molecules transfer RANKL signals from the cell surface receptor to the transcription factors in the nucleus [46]. Our results, presented in Figures 3 and 4, indicated that LABE suppressed intracellular ROS production, TRAP activity, and osteoclast differentiation in RANKL-induced osteoclasts. We conducted further experiments to further investigate the mechanism by which LABE attenuated osteoclast differentiation through intracellular signaling, including MAPK/NF-*κ*B signaling. We evaluated the antiosteoclastogenic effect of LABE on the phosphorylation of ERK, JNK, and p38 in RANKL-induced RAW 264.7 cells. The phosphorylation levels of all three were significantly inhibited in LABE-treated groups (Figure 5(a)). These results suggested that LABE contributed to decreased osteoclast differentiation by reducing activation of MAPK (ERK, JNK, and p38) signaling.

### 3.8. Effect of LABE on RANKL-Induced NF-*κ*B Activation and Translocation

During osteoclastogenesis, RANKL induces and activates various transcription factors, including NF-*κ*B, c-Fos, and NFATc1, which act as positive modulators of osteoclast differentiation. RANKL also induces binding of c-Fos to the NFATc1 promoter region, thereby inducing NFATc1 gene expression [47]. Additionally, RANKL-RANK interaction activates I*κ*B kinase and the NF-*κ*B pathway. NF-*κ*B is localized in the cytoplasm in an inactive form by virtue of its association with a class of inhibitory proteins known as I*κ*Bs [48]. Phosphorylation and proteasomal degradation of I*κ*B proteins permit the translocation of NF-*κ*B from the cytoplasm into the nucleus [49]. Nuclear translocation of NF-*κ*B is essential in osteoclast differentiation. A recent study reported that c-Fos-deficient mice and NF-*κ*B p50 and p52 double-knockout mice developed typical osteopetrosis [48], prompting us to investigate if the presence of LABE attenuated both the phosphorylation of I*κ*B*α* and the translocation of NF-*κ*B into the nucleus (Figure 5(e)). The results indicated that LABE inhibited nuclear translocation of NF-*κ*B by reducing I*κ*B*α* phosphorylation.

### 3.9. Effect of LABE on RANKL-Induced c-Fos and NFATc1 Expression

The transcription factor c-Fos plays an essential role in regulating osteoclastic differentiation and activation by phosphorylated MAPKs. We evaluated the ability of LABE to attenuate RANKL-induced c-Fos expression in RAW cells during osteoclastogenesis [48]. Treatment with LABE downregulated c-Fos protein expression that had been elevated by RANKL stimulation in RAW 264.7 cells (Figure 5(h)). RANKL induces c-Fos expression during osteoclastogenesis, and the binding of c-Fos to the NFATc1 promoter region leads to NFATc1 gene expression as a master regulator of RANKL-induced osteoclast formation and differentiation [23, 49]. We investigated the effect of LABE on downregulation of NFATc1 protein expression in RANKL-mediated osteoclastogenesis (Figure 5(h)) [50]. The expression of NFATc1 protein was highly upregulated by RANKL stimulation. However, treatment with LABE significantly inhibited the upregulation of NFATc1 expression by RANKL stimulation and activation. Our results indicated that the LABE attenuated the expression of c-Fos and NFATc1 transcription factors, which are responsible for osteoclast differentiation. These findings suggested that the suppressive effects of the LABE on osteoclastogenic differentiation were associated with the downregulation of major osteoclastogenic transcription factors such as NF-*κ*B, c-Fos, and NFATc-1.

### 3.10. Effect of LABE on Osteoclast-Specific Gene Expression

By activating a variety of signaling pathways, RANKL induces many osteoclast-specific genes, including calcitonin receptor, TRAP, cathepsin K, and integrin-*β*. NFATc1 also induces many genes downstream of RANKL signaling, ultimately leading to increased osteoclast differentiation [51]. Presently, LABE reduced the induction of osteoclast-specific genes in RANKL-stimulated RAW cells as assessed by western blotting. The expression levels of cathepsin K, TRAP, calcitonin receptor, and integrin *β* were substantially decreased in the presence of LABE (Figures 5(k)–5(o)) Our findings demonstrated that LABE treatment was able to downregulate downstream transcription factors by inhibiting the expression of upstream transcription factors, such as NF-*κ*B, c-Fos, and NFATc1, during RANKL-induced osteoclast differentiation.

### 3.11. Body and Organ Weight Changes of OVX Rats

**Figure 6:**
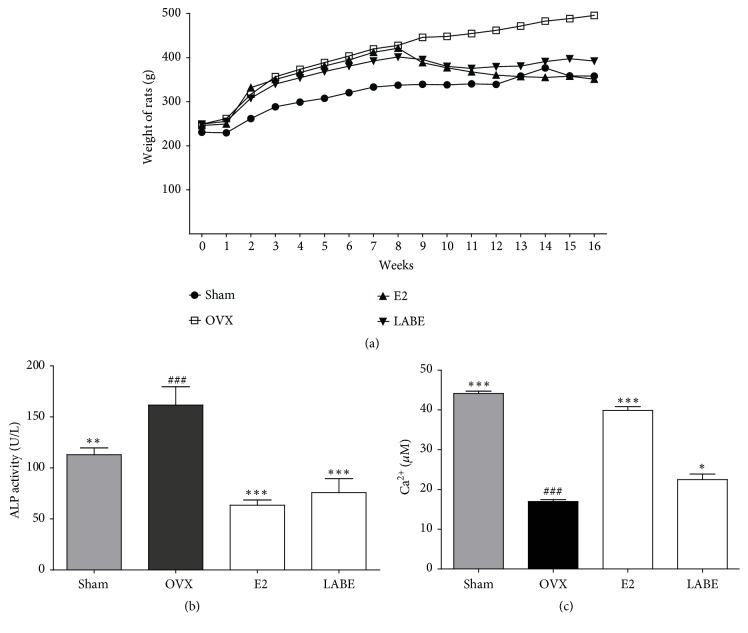
Effect of LABE on osteoporosis. Effects of LABE on (a) body weight, (b) alkaline phosphatase (ALP) activity, and (c) serum calcium level of ovariectomized (OVX) rats. Rat groups include sham-operated (Sham), OVX-control (OVX), E2-treated OVX groups (E2), and LABE 500 mg/kg treated OVX groups (LABE) (N=10/group). Compared are OVX group versus Sham group (###* P *< 0.001) and OVX group versus sample and E2-treated group (*∗P* < 0.05, *∗∗ P* <0.01, and *∗∗∗ P <*0.001).

Our study also included animal experiments to evaluate the effect of LABE on ovariectomy-induced osteoporosis in rats. The weights of liver, kidney, and spleen were measured in OVX rats (Table 3). Organ weight was represented as a percentage of the body weight. Liver, kidney, and spleen weights did not differ significantly between the OVX and Sham groups. During the first 8 weeks of OVX-induced osteoporosis, body weight was increased in all groups, and the body weight of OVX groups including OVX, E2, and LABE was increased compared to that of the Sham group (Figure 6(a)). During the next 8 weeks of LABE oral administration or estradiol injection, however, the body weight gain was substantially reduced in the E2 and LABE groups. The inhibition of body weight gain was greater at a dose of 500 mg/kg LABE.

### 3.12. Serum Biochemical Parameters of OVX Rats

The serum levels of bone turnover markers including ALP and calcium were evaluated (Figure 6(b)). Serum ALP levels in the OVX group were significantly increased compared to those in Sham group, and oral administration of LABE (500 mg/kg) significantly reduced the serum ALP levels as an osteoporosis-related serum marker. The serum calcium levels were decreased in the OVX group, and oral administration of 500 mg/kg LABE restored calcium levels in the OVX group (Figure 6(c)). These results suggested that LABE treatment ameliorated bone loss by inhibiting bone remodeling in OVX rats.

### 3.13. Trabecular Bone Microarchitecture and Cortical Bone Morphology of OVX Rats

**Figure 7:**
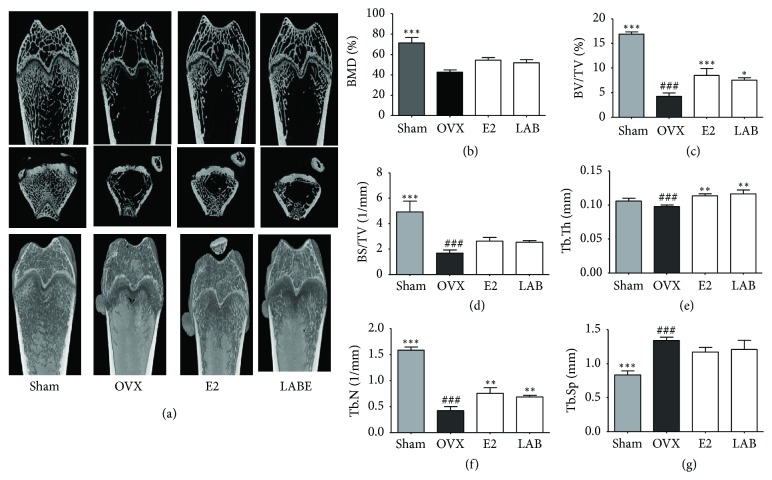
Effects of LABE on trabecular bone parameters in the proximal femurs by micro-CT analysis. (a-f) Trabecular microstructural properties of the right femoral metaphysis evaluated* ex vivo* by micro-CT. (a) BMD, bone mineral density; (b) BV/TV, percent bone volume; (c) BS/TV, bone surface density; (d) Tb.N, trabecular number; (e) Tb.Th, trabecular thickness; (f) Tb.Sp, trabecular separation. (g) Coronal micro-CT images of rat medial-proximal femur are shown. (f) 3D images were taken by micro-CT. Rat groups include sham-operated (Sham), OVX-control (OVX), E2-treated OVX groups (E2), and LABE 500 mg/kg treated OVX groups (500 mg/kg; N = 10/group). Compared are OVX group versus Sham group (###* P *< 0.001) and OVX group versus sample and E2-treated group (*∗P* < 0.05, *∗∗ P* <0.01, and *∗∗∗ P <*0.001).

To evaluate the effect of LABE on the proximal femur changes induced by ovariectomy, the trabecular bone microarchitecture in the proximal femurs was analyzed by micro-CT (Figure 7). Quantitative analyses of BMD, BV/TV, Tb.Th, Tb.Sp, Tb.N, BS/TV, and trabecular bone microarchitecture were measured. 2D and 3D images of proximal femurs taken by micro-CT showed differences in trabecular microarchitecture among the treatment groups (Figure 7). Bone microarchitecture parameters did not differ significantly between the OVX group and the LABE-supplemented group. As shown in the images of the femoral trabecular bone microarchitecture (Figure 7(a)), the OVX-induced group showed significant changes in all trabecular microstructural parameters in the proximal femoral metaphysis. Bone cortical thickness and bone mass were decreased, and the trabecular bone microarchitecture was significantly impaired in the OVX group compared to the Sham group. Conversely, the administration of LABE significantly improved the bone microstructure damage and the decreased bone cortical thickness and bone mass. The LABE-treated group showed significantly higher percent of BV/TV, BS/TV, Tb.N, and Tb.Th and significantly lower Tb.Sp compared to the OVX group (Figures 7(c)–7(g)). The BV/TV, Tb.Th, and Tb.N values were significantly greater in the LABE and E2 groups than in the OVX group. LABE administration enhanced BV/TV, BS/TV, Tb.N, and Tb.Th levels and decreased Tb.Sp levels in trabecular bones of treated rats compared to those from OVX rats. These results suggested that LABE administration can prevent the OVX-induced changes in these bone microstructure parameters and help to maintain trabecular bone microarchitecture.

### 3.14. H&E and TRAP Staining of Bone Tissue from OVX Rats

**Figure 8:**
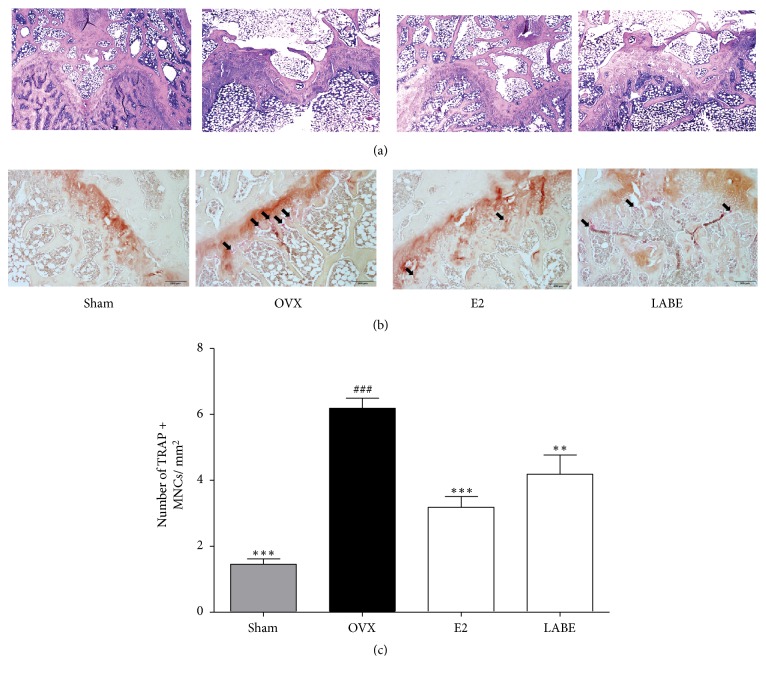
H&E and TRAP staining of the trabecular bone tissue from the femur. (a) H&E staining shows trabecular bone and (b) TRAP staining indicates TRAP in osteoclasts (black arrows) in trabecular bone. (c) The number of TRAP-positive multinucleated cells (MNCs) per mm2 of bone surface. Compared are OVX group versus Sham group (###* P *< 0.001) and OVX group versus sample and E2-treated group (*∗P* < 0.05, *∗∗ P* <0.01, and *∗∗∗ P <*0.001). Rat groups include sham-operated (Sham), OVX-control (OVX), E2-treated OVX groups (E2), and LABE 500 mg/kg treated OVX groups (500 mg/kg; N = 10/group).

H&E (Figure 8(a)) and TRAP (Figure 8(b)) staining of the sectional femoral bone tissue was performed to confirm the protective effect of LABE treatment on OVX-induced bone loss. The results with femoral bone tissues further corroborated the results obtained by micro-CT. In the Sham group, H&E staining revealed that the trabeculae exhibited densely arranged structures with small interspaces in the metaphysis and epiphysis of the proximal femoral. The OVX group showed thinning and sparse trabeculae with poor continuity and alignment, while LABE treatment resulted in improved trabecular bone integrity compared to that of the OVX group, suggesting a role for LABE in protecting trabecular bone from ovariectomy-induced bone loss. TRAP staining to determine osteoclast differentiation in OVX rats was performed. TRAP, which has been proposed as a cytochemical marker for osteoclasts, was increased during osteoclastogenesis. Compared to the OVX group, the LABE-treated group displayed a decreased number of TRAP-positive multinucleated cells at the growth plates of the long bones (Figures 8(b) and 8(c)). These TRAP staining results were in accordance with those of micro-CT and H&E staining. H&E and TRAP staining suggested that LABE treatment inhibited OVX-induced bone loss and the formation of TRAP-positive osteoclasts on the bone surfaces.

## 4. Discussion

Osteoclasts play a very important role in diseases involving excessive bone resorption. It is therefore rational to consider osteoclasts an effective target for the prevention, treatment, and control of these osteolytic diseases. RANKL promotes osteoclast differentiation. Given this, RANKL-induced osteoclastogenesis plays an important role in the prevention of several bone diseases.

In the present study, we demonstrated the effects of 70% ethanol LABE in attenuating RANKL-induced osteoclastic differentiation in RAW 264.7 murine macrophage cells and ovariectomy-induced osteoporosis in rats.

The effect of LABE on antioxidant efficiency and ROS inhibition activity was evaluated. Four anthocyanin compounds were identified in LABE by HPLC analysis (Figure 1 and Table 1). The LABE also contained considerable amounts of total phenolic and flavonoid compounds (Table 2). Black rice (*O. sativa L*.) contains anthocyanins, which act as major bioactive compounds [52]. Total anthocyanin, phenolic, and flavonoid components were reported to be positively correlated with antioxidant activity [28, 30, 31]. Additionally, anthocyanins found in black rice are responsible for antiadipogenic activities, and other black rice components, such as ferulic acid and coumaric acid, contribute to the proosteogenic effects [15]. Antioxidants protect the human body from the action of free radicals and ROS. Cells possess antioxidant networks that scavenge excessive ROS [53]. LABE exhibited strong antioxidant potency, as shown by its high DPPH and ABTS radical scavenging activity and reducing power (Figure 2), which can contribute to the inhibition of ROS production. The inhibitory effects of various flavonoid compounds, including quercetin [54], rutin [55], and luteolin [56], on osteoclast differentiation have been described, as have the antiosteoporosis effects of anthocyanin and anthocyanin compounds from bilberry and black currant, which inhibited osteoclast formation from osteoclast precursor RAW 264.7 cells [57].

Binding of RANKL to RANK on cell surfaces of osteoclast precursors induces the generation of intracellular ROS. The increased ROS can facilitate osteoclast bone resorption and osteoclast differentiation during the process of bone resorption [34, 36–40, 54].

Our results demonstrated that LABE significantly suppressed the intracellular ROS levels generated in RAW cells induced using H_2_O_2_ (50 *μ*M) or RANKL (50 ng/mL) (Figure 3), which subsequently suppressed the activation of downstream signaling in RANKL-induced osteoclasts.

In RANKL-treated RAW cells, RANKL-RANK binding promotes the differentiation of osteoclast precursor cells into TRAP-positive multinuclear osteoclasts [37]. The expression of TRAP is highly increased during osteoclast differentiation and formation. The present results of the TRAP activity and staining assay indicated that LABE significantly suppressed TRAP activity in TRAP-positive multinucleate osteoclasts in a dose-dependent manner (10-1000 ppm) (Figures 4(a), 4(b), and 4(c)).

RANKL binding-induced intracellular ROS also stimulate RANKL-mediated signaling in osteoclast precursor bone marrow macrophages [34, 41]. The generated ROS activate ERK, JNK, and p38 MARKs, and the activation of these MARK pathways leads to the translocation of c-Fos and NFATc1 into the nucleus. In this study, LABE inhibited RANKL-induced ROS generation and downregulated JNK, ERK, and p38 phosphorylation in RAW 264.7 cells (Figures 3(a), 3(b), 5(a)–5(d)).

These results indicate that LABE can downregulate MAPK expression by inhibiting intracellular production of ROS, which play a crucial role as secondary messengers in the signaling pathway of RANKL osteoclast differentiation.

I*κ*B kinase and the NF-*κ*B pathway are activated by RANKL-RANK interaction, and NF-*κ*B is essential for osteoclast differentiation and activation [48]. Phosphorylation and proteasomal degradation of I*κ*B proteins permit the translocation of NF-*κ*B from the cytoplasm into the nucleus [49], a process that is essential to active gene transcripts associated with osteoclast differentiation [49, 58]. In this study, LABE inhibited I*κ*B phosphorylation and nuclear translocation of NF-*κ*B, both of which are associated with the downstream signal activation of RANKL-induced osteoclastic differentiation (Figures 5(e)–5(g)).

Both c-Fos and NFATc1 are key transcriptional factors. They are induced by RANKL and are pivotal in RANKL-induced osteoclastogenesis [43, 51]. Binding of c-Fos to NFATc1 mediated by RANKL signaling leads to the expression of NFATc1. Presently, LABE treatment significantly downregulated the protein expression of RANKL-induced c-Fos and NFATc1 (Figures 5(h)–5(j)). It is well established that NFATc1, a master regulator of osteoclast differentiation, regulates a number of osteoclast-specific genes including cathepsin K, TRAP, integrin *β*3, c-Src, and the calcitonin receptor [51]. Our results confirmed that the expressions of cathepsin K, TRAP, calcitonin receptor, and integrin *β*3 were upregulated by RANKL, and treatment with LABE significantly downregulated the expressions of these genes (Figures 5(k)–5(o)).

According to our results, the suppressive effect of LABE on ROS generation can lead to reduced activation of MAPK signaling and inhibited expression of cathepsin K, TRAP, integrin *β*3, and calcitonin receptor by inhibiting the JNK/ERK/p38 NF-*κ*B-mediated c-Fos/NFATc1 signaling pathways. Previous research has demonstrated that black rice can stimulate osteogenesis and enhance osteogenic differentiation through increased osteogenic gene expression in C3H10T1/2 cells [14]. The antioxidative nutrients and phytochemicals in fruits and vegetables have been shown to improve bone health by scavenging ROS [59].

The OVX rat model we used exhibits characteristics similar to those of human postmenopausal osteoporosis. This model is the current Food and Drug Administration approved model for the investigation of menopausal bone changes and is commonly used in the laboratory [60, 61]. Our* in vivo* study demonstrated that oral administration of LABE protected against ovariectomy-induced osteoporosis in rats by significantly blocking alterations in bone architectural parameters and serum bone turnover markers.

LABE and 7*β*-estradiol administration reduced rat body weight gain (Figure 6(a)), suggesting that LABE administration exerts a beneficial effect on osteoporosis and thus reduces weight gain by ovariectomy. 7*β*-Estradiol is important in the regulation of energy metabolism and body weight. Therefore, ovariectomy results in body weight gain, while 7*β*-estradiol administration results in a reversal of weight gain [62].

Previous studies of postmenopausal women and experimental animals have reported correlations between blood ALP and calcium level [63] and trabecular bone microarchitecture [64, 65]. Kim* et al.* reported that black rice ethanol extract stimulated osteogenesis, enhancing the mRNA expression of runt‐related transcription factor 2 and ALP. This, in turn, increased ALP protein expression and cellular enzyme activity, thus elevating intracellular calcium deposition [15]. Bone-formation biomarkers, including ALP and calcium, have been used to measure the effect of bone remodeling on various drug treatments. Presently, the level of ALP, an osteoporosis-related serum marker, was increased in the OVX group, while oral administration of LABE (500 mg/kg) for 8 weeks significantly reduced the serum ALP levels compared to OVX group without the influence of hormones, such as estrogen (Figure 5(b)). LABE administration also improved the decreased serum calcium levels observed in the OVX group (Figure 6(c)). These results indicate that LABE treatment can ameliorate bone loss by inhibiting bone remodeling in OVX rats.

The micro-CT-based image analysis of proximal femurs revealed that the trabecular bone microarchitecture properties and parameters were significantly different between the OVX and LABE-administrated groups (Figure 7). OVX in rats caused significant changes in trabecular microstructural properties and parameters in the proximal femoral metaphysis. Imaging of proximal femurs revealed that the OVX group had decreased cortical thickness and bone mass and considerably impaired trabecular bone microarchitecture compared to the Sham group. These OVX-induced changes in trabecular microstructure were significantly improved by the oral administration of LABE. Our findings indicate a significant antiosteoporosis effect in the LABE-treated group compared to that in the OVX group.

The micro-CT images accurately represent bone microstructural parameters for quantitative assessments. The BS/TV and Tb.Th values were significantly higher in the LABE and E2 groups than in the OVX group. The oral administration of LABE significantly enhanced BV/TV, BS/TV, Tb.N, and Tb.Th levels and decreased Tb.Sp levels in the femur trabecular bone microarchitecture compared to levels in OVX rats. Loss of estrogen in OVX rats inhibits osteoblast differentiation and accelerates bone remodeling with a predominance of bone resorption compared to bone formation [66]. Our results suggest that LABE treatment can prevent the alteration of these parameters induced by OVX and maintain the trabecular bone microstructure (Figure 7).

In H&E staining (Figure 8(a)), rats in the OVX group showed sparse and thinning trabeculae with loss of connectivity. Administration of LABE and estradiol significantly protected trabecular bones from ovariectomy-induced bone loss and changes in rats. TRAP, a cytochemical marker for osteoclasts, was increased during osteoclastogenesis and osteoclast differentiation [61]. TRAP was dyed red by TRAP staining, and TRAP-positive osteoclast cells appeared red (Figure 8(b)). TRAP and TRAP-positive osteoclasts (Figures 8(b) and 8(c)) were induced on trabecular bone surfaces to a greater extent with the activation of OVX-induced osteoclastogenesis in the OVX group than in the LABE group, while LABE treatment decreased TRAP-positive osteoclasts. This indicated that LABE treatment inhibits osteoclast differentiation and bone resorption.

## 5. Conclusion

Our* in vitro* findings demonstrated that LABE can inhibit ROS production and the activation of MAPKs and NF-*κ*B, resulting in downregulated expression of c-Fos and NFATc1. This consequently suppressed the expression of osteoclast-specific genes, including cathepsin K, TRAP, calcitonin receptor, and integrin *β*3.* In vivo*, the oral administration of LABE improved changes in bone microarchitectural parameters and properties associated with ovariectomy-induced osteoporosis in rats.

## Figures and Tables

**Table 1:** Content of anthocyanin compounds in LABE.

Peak	Compounds	Content (mg/g)
1	Cyanidin-3,5-diglucoside	0.0858
2	Cyanidin-3-glucoside	1.0592
3	Cyanidin-3-rutinoside	0.0123
4	Peonidin-3-glucoside	0.0421

**Table 2:** Total phenolic and flavonoid content and yield of LABE.

Samples	Total phenolic (mg GAE†/100g of dry mass)	Total flavonoid (mg CE‡/100g of dry mass)	Yield (%)
LABE	168.7 ± 2.70	214.38± 16.77	7.44

All data are expressed as mean ± standard deviation (n=3).

†GAE: gallic acid equivalent; ‡CE: catechin equivalent

**Table 3:** Liver, kidney, and spleen weight (%, organ/body weight) in mice with different treatments.

	Sham	OVX	E2	LABE
Liver	9.65±1.62^a^	12.17±2.05^b^	10.13±0.88^ab^	9.92±1.63^a^
Kidney	1.91±0.05^a^	2.01±0.17^ab^	2.30±0.27^b^	1.94±0.33^ab^
Spleen	0.46±0.04^a^	0.7±0.17^b^	0.52±0.06^a^	0.57±0.05^ab^

One-way analysis of variance (ANOVA) test followed by multiple-comparison *t*-test. P value: <0.05.

^a,b^Values are means ± standard error (n=10, each group).

Means within the same column which have no common superscript letters are significantly different from each other (*P *<0.05).

## Data Availability

The data used to support the findings of this study are available from the corresponding author upon request.
